# STEAP3 promotes cancer cell proliferation by facilitating nuclear trafficking of EGFR to enhance RAC1-ERK-STAT3 signaling in hepatocellular carcinoma

**DOI:** 10.1038/s41419-021-04329-9

**Published:** 2021-11-05

**Authors:** Li-Li Wang, Jie Luo, Zhang-Hai He, Ye-Qing Liu, Hai-Gang Li, Dan Xie, Mu-Yan Cai

**Affiliations:** 1 State Key Laboratory of Oncology in South China; Collaborative Innovation Center for Cancer Medicine; Sun Yat-Sen University Cancer Center, Guangzhou, Guangdong China; 2 Department of Pathology, Sun Yat-Sen Memorial Hospital, Sun Yat-Sen University, Guangzhou, Guangdong China; 3 Department of Pathology, Sun Yat-Sen University Cancer Center, Guangzhou, Guangdong China

**Keywords:** Oncogenes, Tumour biomarkers

## Abstract

STEAP3 (Six-transmembrane epithelial antigen of the prostate 3, TSAP6, dudulin-2) has been reported to be involved in tumor progression in human malignancies. Nevertheless, how it participates in the progression of human cancers, especially HCC, is still unknown. In the present study, we found that STEAP3 was aberrantly overexpressed in the nuclei of HCC cells. In a large cohort of clinical HCC tissues, high expression level of nuclear STEAP3 was positively associated with tumor differentiation and poor prognosis (*p* < 0.001), and it was an independent prognostic factor for HCC patients. In HCC cell lines, nuclear expression of STEAP3 significantly promoted HCC cells proliferation by promoting stemness phenotype and cell cycle progression via RAC1-ERK-STAT3 and RAC1-JNK-STAT6 signaling axes. Through upregulating the expression and nuclear trafficking of EGFR, STEAP3 participated in regulating EGFR-mediated STAT3 transactivity in a manner of positive feedback. In summary, our findings support that nuclear expression of STEAP3 plays a critical oncogenic role in the progression of HCC via modulation on EGFR and intracellular signaling, and it could be a candidate for prognostic marker and therapeutic target in HCC.

## Introduction

Primary liver cancer is the sixth most common cancer and the fourth leading cause of cancer-related death worldwide, with 841,080 new cases and 781,631 death in 2018. Hepatocellular carcinoma (HCC) accounts for up to 85% of all primary liver cancer cases [1, 2]. Early diagnosis of HCC is difficult, and the prognoses of HCC patients haven’t been significantly improved due to limited effective therapies, with a 5-year survival rate of about 18%. 5-year recurrence rates can reach >70% even in early-stage HCC patients [3, 4]. Apparently, the high incidence and mortality of HCC call for more effective therapeutic strategies.

STEAP3 is a member of the STEAP family and is composed of a six-transmembrane domain at the COOH-terminal domain and a cytoplasmic N-terminal oxidoreductase domain, which is essential for iron and copper uptake [5, 6]. STEAP3 mRNA is highly expressed in the liver, bone marrow, placenta, skeletal muscle, and heart [5, 6]. In light of the fact that its depletion leads to hypochromic microcytic anemia, STEAP3 plays an important role in the hematopoiesis, especially in erythroid precursors, by regulating iron metabolism [7, 8]. STEAP3 contains a functional p53-binding site in its promoter and can be upregulated following p53 activation to enhance cell death in myeloid leukemia cell line and breast cancer cells [9, 10]. By interacting with Nix, a pro-apoptotic Bcl-2 family member, and Myt1 kinase, a negative regulator of the G2/M transition, STEAP3 overexpression promotes apoptosis and inhibits G2/M transition in cell cycle progression [6, 11, 12]. Despite being considered as a tumor suppressor, STEAP3 is involved in the progression of a series of human malignancies. STEAP3 is overexpressed in prostate cancer and metastatic high-grade serous carcinoma [13, 14]. STEAP3 mRNA level is upregulated to maintain tumor proliferation under hypoferric conditions in colorectal carcinoma [15].

Liver is at the center of iron homeostasis regulation [16]. Through regulating inflammatory responses as well as apoptosis, STEAP3 mediates hepatic ischemia-reperfusion injury via TAK1-dependent activation of the JNK/p38 pathways [17]. Due to its expression being remarkably diminished in HCC nodules compared with cirrhotic peritumoral tissues, STEAP3 was reported to be a marker of the transition from cirrhosis to hepatocellular carcinoma [18, 19]. However, how STEAP3 participates in the progression of HCC is still unknown. Herein, for the first time, we found that an aberrant nuclear expression pattern of STEAP3 which promotes HCC cell proliferation by interacting with EGFR and enhancing EGFR-RAC1-ERK-STAT3 and RAC1-JNK-STAT6 signaling, and it could be employed as a novel prognostic marker and/or effective therapeutic target for human HCC.

## Material and methods

### Cell culture and reagents

Human HCC cell lines, PLC/PRF/5, QGY-7701, HepG2, Hep3B, SNU449, and Huh-7, were purchased from ATCC, authenticated by STR profiling, and tested for mycoplasma contamination. The cell lines were maintained in DMEM (Gibco, Thermo Fisher Scientific, Grand Island, NY, USA) supplemented with 10% fetal bovine serum (Gibco, Thermo Fisher Scientific, Grand Island, NY, USA), 100 units/mL penicillin, and 100 μg/mL streptomycin in a 5% CO_2_ humidified incubator at 37 °C. DMSO was purchased from Sigma-Aldrich (St. Louis, MO, USA). Primary antibodies and chemicals used in this study were listed in Supplementary Table 1 and Table 2.

### Patients

In this study, formalin-fixed, paraffin-embedded pathological specimens from 200 patients who underwent initial surgical resection between 2003 and 2006 were obtained from the archives of the Department of Pathology of the First Affiliated Hospital of Sun Yat-Sen University (Guangzhou, China). Patients with follow-up data were selected only if they had been given an explicit pathological diagnosis, underwent primary and curative resection, and received no preoperative anticancer treatment. The tumor stage was defined according to the TNM classification of malignant tumors (UICC). Written informed consent was obtained from all patients.

### Immunohistochemistry (IHC) and selection of cut-point score

HCC tissue slides were incubated with primary antibodies overnight at 4 °C. Immunostaining was performed using the EnVision FLEX Systems (Dako, Santa Clara, CA, USA). Negative control was obtained by replacing the primary antibody with normal goat IgG. The histopathology evaluation in each case was conducted independently by two pathologists in a blind manner. Scores were assigned in a semiquantitative method. In brief, each TMA spot was assigned an intensity score from 0–3 (I0, I1–3). Then, the proportion of tumor cells of that intensity was divided by the total number of tumor cells and recorded in 5% increments from 0 to 100 (P0, P1–3). The final *H*-score (range 0–300) was determined by adding the sum of the scores obtained for each intensity and the proportion of the area stained (*H* score = [I1 × P1] + [I2 × P2] + [I3 × P3]). X-tile plots were used for the assessment of expression and optimization of cut-point based on the outcome by Miller–Siegmund *P*-value correction.

### Western blot

Whole-cell lysates were prepared with a proteinase inhibitor cocktail and phosphatase inhibitor cocktail (Roche, Alameda, CA, USA). Protein concentrations were determined using the BCA protein assay (Thermo Fisher Scientific, Grand Island, NY, USA). Equal amounts of proteins were subjected to 10% SDS-polyacrylamide gel electrophoresis and transferred to PVDF membranes (Millipore, Danvers, MA, USA). Membranes were blocked with 5% non-fat dry milk containing 0.1% Triton X-100 at room temperature and blotted with primary antibodies overnight at 4 °C. Signals were visualized by chemiluminescence (Thermo Fisher Scientific, Grand Island, NY, USA). Goat anti-rabbit IgG HRP-linked antibody (1:5000) and goat anti-mouse IgG HRP-linked antibody (1:5000) were from Proteintech (Rosemont, IL, USA).

### Construction of the recombinant lentiviral vector

Lv105-STEAP3 and Lv105 control vector, and pGFP-ShLenti-STEAP3 and pGFP-ShLenti control vector were purchased from GeneCopoeia (Rockville, MD, USA). Stocks of virus were generated in 293-T cell line using Lenti-vpak packaging kit (OriGene, Cambridge, MA, USA). Stably infected cells were selected using puromycin (InvivoGen, CA, USA).

### Cell Counting Kit-8 (CCK8) assay

Cells were seeded in 96-well plates at a density of 1,000 cells per well. CCK8 (MedChemExpress, NJ, USA) was added to each well and incubated for 1 h at 37 °C. The optical density (OD) was measured at wavelengths of 450 nm and 650 nm using a microplate reader at different time periods. The calibrated OD450 value was calculated as OD450-OD650.

### Colony formation assay

Cells were seeded in 6-well plates at a density of 300 cells per well. The colonies were visualized by staining with crystal violet after 2 weeks.

### Sphere formation assay

Cells were suspended in DMEM/F12 (Gibco, Thermo Fisher Scientific, Grand Island, NY, USA) supplemented with 20 ng/mL EGF (Gibco, Thermo Fisher Scientific, Grand Island, NY, USA), 20 ng/mL bFGF (Gibco, Thermo Fisher Scientific, Grand Island, NY, USA), and N-2 supplement (Gibco, Thermo Fisher Scientific, Grand Island, NY, USA), and plated into ultra-low attachment 6-well plates (Corning, Corning, NY, USA) at a density of 300 cells/well for 10 days. Spheroids were counted under an Olympus CKX41 light microscope (Olympus, Tokyo, Japan).

### Flow cytometry

Cells were seeded in 6-well plates, synchronized with serum-free medium for 24 h, and cultured in a medium containing 10% fetal bovine serum for the indicated period of time. For cell cycle analysis, cells were harvested and fixed in cold 70% ethanol and analyzed by propidium iodide (PI) staining using the Cell Cycle and Apoptosis Analysis Kit (Beyotime Biotechnology, Shanghai, China).

### Transwell assay

Cells were starved with serum-free medium for 24 h and seeded at a density of 10^5^ in the upper chamber of Transwell chambers with 8 μm pore polycarbonate membranes (Corning, NY, USA) in serum-free medium. Medium containing 20% FBS was added to the lower chambers. After incubating for 12 h, cells were stained with crystal violet. Cells crossed through the pores to the lower surface of the filter were counted in 20 high-power fields (200×) under a microscope.

### Tumor growth in xenografts

3 × 10^6^ of Hep3B-STEAP3-Sh2 and Hep3B-ShControl cells were injected into the flanks of two groups of 4-week-old male BALB/c nude mice, respectively. Three weeks after subcutaneous inoculation, mice were euthanized, and tumor xenografts were instantly removed and weighed. The sample size was established based on previous work with the animal model. No animal was excluded from the analysis. No randomization was used to allocate samples/animals to experimental groups. The investigator was not blinded to the group allocation of samples/animals.

### Laser scan confocal microscope

Cells were seeded on coverslips and allowed to adhere for the indicated period of time. Cells were fixed and permeabilized with cold menthol for 15 min at −20 °C, then blocked with 5% BSA in PBS containing 3% Tween-20 for 1 h at room temperature. Cells were incubated with primary antibodies overnight at 4 °C. After washing, cells were then incubated with a secondary antibody for 1 h at room temperature. Images were captured using LSM 880 with Airyscan (Zeiss, German) and analyzed using ZEN 2.6 software (Zeiss, German). Anti-rabbit IgG H&L (Alexa Fluor® 647) (1:1000) and anti-mouse IgG H&L (Alexa Fluor® 594) (1:1000) were from Abcam (Cambridge, MA, USA).

### Co-immunoprecipitation (Co-IP)

Cell lysates preparation and immunoprecipitation were conducted using Crosslink Magnetic IP/Co-IP Kit (Thermo Fisher Scientific, Grand Island, NY, USA) according to the manufacturer’s instructions. The cell lysate was incubated with primary antibodies on a rotator overnight at 4 °C, then subjected to western blot for analysis.

### Real-time RT-PCR

Total RNA from each sample was quantified by the NanoDrop ND-2000 (Thermo Fisher Scientific, Grand Island, NY, USA) and converted to cDNA using PrimeScript™ RT Master Mix (Takara, Kyoto, Japan). Real-time PCR was performed using GoTaq® qPCR Master Mix (Promega, Madison, WI, USA) on a CFX96 Touch Real-Time PCR Detection System (Bio-Rad, California, USA). Primers used in this study were listed in Supplementary Table 3.

### Statistical analysis

For survival analysis, the optimal cut-point for STEAP3 expression was obtained using X-tile software version 3.6.1 (Yale University School of Medicine, New Haven, CT). The correlation between STEAP3 expression and clinicopathological features of HCC patients was analyzed using the *χ*2 test or Fisher’s exact test. For univariate survival analysis, survival curves were obtained using the Kaplan–Meier method. A two-sided Student’s *t-*test was performed to analyze the statistical significance between two pre-selected groups. *P*-values < 0.05 were considered statistically significant.

## Results

### Nuclear expression of STEAP3 is an independent prognostic factor for the poor outcome of HCC patients

**Fig. 1:**
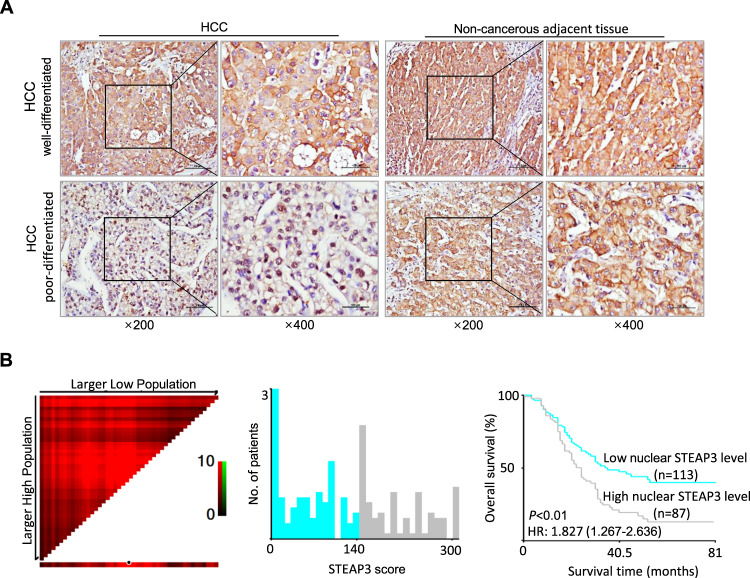
Nuclear expression of STEAP3 is an independent prognostic factor for poor survival of HCC patients. **A** Expression levels of STEAP3 were examined by IHC in well- and poor-differentiated HCC tissues (left). Representative images show that STEAP3 was located in the cytoplasm in non-cancerous adjacent tissue (right) and well-differentiated HCC, but distributed in the nuclei in poor-differentiated HCC. Scale bar, 100 μm. **B** X-tile plots were employed to determine the cut-point for the nuclear expression of STEAP3 in a large cohort of 200 HCCs. The cut-point was defined as *H*-score > 140, and highlighted by the black circle in the horizontal axis (left) and demonstrated on a histogram of the cohort (middle). The *χ*2 log-rank values were defined by Kaplan–Meier method when using the cut-point to divide the cohort into low and high populations, and the *p*-value was determined (right) (*p* < 0.01).

To explore the role of STEAP3 in the progression of HCC, we first examined its expression dynamics by immunohistochemistry in a large cohort of clinical HCC and paired non-cancerous adjacent liver tissues. As reported [18], we found that non-cancerous adjacent liver tissues and well-developed HCC tissues exhibited strong cytoplasm expression of STEAP3, while poor-differentiated HCC tissues showed low STEAP3 expression in the cytoplasm. However, aberrant high levels of STEAP3 were observed in the nuclei of 55 poor-differentiated HCC, which were absent in non-cancerous adjacent liver tissues and most of well-differentiated HCC tissues (Fig. 1A). Based on X-tile plots, the cut-point for high nuclear expression of STEAP3 was defined when the *H*-score was above 140 (Fig. 1B). Correlation analysis demonstrated that high nuclear expression of STEAP3 was positively associated with tumor differentiation and relapse (*p* < 0.001, Table 1). The mean disease-free survival time for HCC patients with high nuclear expression of STEAP3 was 29.5 months compared with a survival time of 46.4 months for patients with low nuclear expression of STEAP3 (*p* < 0.01, log-rank test, Table 2). Furthermore, nuclear STEAP3 expression is an independent prognostic factor for poor survival of HCC patients (*p* < 0.05, multivariate Cox regression).

**Table 1:** Correlation of nuclear expression of STEAP3 with patients’ clinicopathological features in 200 primary hepatocellular carcinomas.

**Variable**	**STEAP3 protein**			
	**All cases**	**Low expression**	**High expression**	***P*-value**^a^
*Age (years)*				0.125
≤ 47.9^b^	98	50 (51.0%)	48 (49.0%)	
> 47.9	102	63 (61.8%)	39 (38.2%)	
*Sex*				0.646
Female	34	18 (52.9%)	16 (47.1%)	
Male	166	95 (57.2%)	71 (42.8%)	
*HBsAg*				0.247
Negative	47	30 (63.8%)	17 (36.2%)	
Positive	153	83 (54.2%)	70 (45.8%)	
*AFP (ng/ml)*				0.108
≤ 20	65	42 (64.6%)	23 (35.4%)	
> 20	135	71 (52.6%)	64 (47.4%)	
*Liver cirrhosis*				0.811
No	74	41 (55.4%)	33 (44.6%)	
Yes	126	72 (57.1%)	54 (42.9%)	
*Tumor size (cm)*				0.058
≤ 5	55	37 (67.3%)	18 (32.7%)	
> 5	145	76 (52.4%)	69 (47.6%)	
*Tumor multiplicity*				0.608
Single	119	69 (58.0%)	50 (42.0%)	
Multiple	81	44 (54.3%)	37 (45.7%)	
*Differentiation*				0.000
Well-moderate	143	111 (77.6%)	32 (22.4%)	
Poor-undifferentiated	57	2 (3.5%)	55 (96.5%)	
*Stage*				0.176
I-II	82	51 (62.2%)	31 (37.8%)	
III- IV	118	62 (52.5%)	56 (47.5%)	
*Vascular invasion*				0.077
Absent	97	61 (62.9%)	36 (37.1%)	
Present	103	52 (50.5%)	51 (49.5%)	
*Relapse*				0.000
Absent	102	78 (76.5%)	24 (23.5%)	
Present	98	35 (35.7%)	63 (64.3%)	

^a^Chi-square test; ^b^Mean age.

HBsAg hepatitis B surface antigen; AFP alpha-fetoprotein.

**Table 2:** Univariate analysis of nuclear expression of STEAP3 and clinicopathologic variables in 200 patients with primary hepatocellular carcinomas (log-rank test).

Variables	All cases	RR (95% CI)	*P*-value
*Age (years)*			0.655
≤ 47.9^a^	98	1.0	
> 47.9	102	1.085 (0.759–1.550)	
*Sex*			0.049
Female	34	1.0	
Male	166	1.687 (0.996-2.856)	
*HBsAg*			0.684
Negative	47	1.0	
Positive	153	1.093 (0.712–1.678)	
*AFP (ng/ml)*			0.000
≤ 20	65	1.0	
> 20	135	2.196 (1.441–3.347)	
*Liver cirrhosis*			0.584
No	74	0.902 (0.625–1.303)	
Yes	126	1.0	
*Tumor size (cm)*			0.000
≤ 5	55	1.0	
> 5	145	6.107 (3.520–10.597)	
*Tumor multiplicity*			0.000
Single	119	1.0	
Multiple	81	3.396 (2.349–4.911)	
*Differentiation*			0.037
Well-moderate	143	1.0	
Poor-undifferentiated	57	1.483 (1.023–2.149)	
Stage			0.000
I–II	82	1.0	
III–IV	118	5.667 (3.622–8.866)	
*Vascular invasion*			0.000
Absent	97	1.0	
Present	103	5.164 (3.409–7.823)	
*Relapse*			0.001
Absent	102	1.0	
Present	98	1.878 (1.299–2.717)	
*STEAP3 expression*			0.001
Low	113	1.0	
High	87	1.866 (1.302–2.676)	

^a^Mean age; CI, confidence interval.

HBsAg hepatitis B surface antigen; AFP alpha-fetoprotein.

### Enhanced nuclear expression of STEAP3 promotes cancer cell proliferation in HCC

**Fig. 2:**
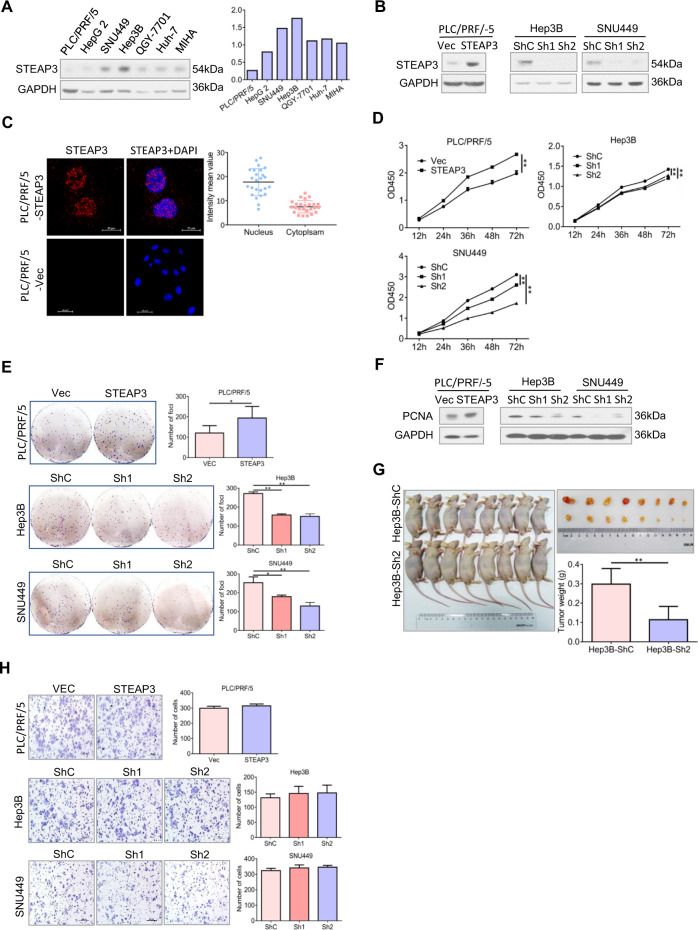
Enhanced nuclear expression of STEAP3 promotes cancer cell proliferation in HCC. **A** Expression levels of STEAP3 were examined by western blot in HCC cell lines (PLC/PRF/5, HepG2, SNU449, Hep3B, QGY-7701, and Huh-7) and immortalized hepatocyte (MIHA). **B** STEAP3 was stably overexpressed in PLC/PRF/5-STEAP3 or knocked down in Hep3B-ShSTEAP3 and SNU449-ShSTEAP3, and verified by western blot. **C** Subcellular distribution of STEAP3 (red) was examined by IF in PLC/PRF/5-STEAP3 and control cells. Fluorescence intensity mean values of STEAP3 in the nuclei and cytoplasm were measured in randomly selected 25 cells in PLC/PRF/5-STEAP3. Nuclei were visualized by DAPI (blue). Scale bar, 20 μm. *p* < 0.01. **D** Cell viability was examined by CCK8 assays. Data are presented as means ± SD of three independent experiments in triplicates. **p < 0.01. **E** Cell proliferation was monitored by colony formation. Data are presented as means ± SD of colony foci of three independent experiments. *p < 0.05, **p < 0.01. **F** PCNA levels were examined by western blot. **G** A subcutaneous xenograft tumor mouse model was carried out by injecting Hep3B-ShC and Hep3B-ShSTEAP3-2 into two groups of nude mice, respectively (left). Tumor masses were weighed (right). Data are presented as means ± SD. **p < 0.01. **H** Cell mobility was determined by transwell chamber assays. Data are showed as mean ± SD of three independent experiments. Scale bar, 200 μm. *p* > 0.05.

To validate the functional significance of aberrant nuclear expression of STEAP3 as observed in HCC tissues, we first examined its expression level in HCC cell lines and immortalized hepatocyte MIHA (Fig. 2A), and accordingly established HCC cell lines in which STEAP3 was stably upregulated (PLC/PRF/5-STEAP3) or knockdown (Hep3B-ShSTEAP3 and SNU449-ShSTEAP3) (Fig. 2B). Then, by examining its subcellular localization, an enhanced nuclear distribution of STEAP3 was confirmed in PLC/PRF/5-STEAP3 (*p* < 0.01, Fig. 2C). Subsequently, functional assays were adopted to explore the effects of altered STEAP3 expression on cell proliferation. Compared to paired control cells, PLC/PRF/5-STEAP3 exhibited increased cell viability (*p* < 0.01, Fig. 2D) and colony formation (*p* < 0.05, Fig. 2E). Meanwhile, Hep3B-ShSTEAP3 and SNU449-ShSTEAP3 showed decreased cell viability (*p* < 0.01, Fig. 2D) and colony formation (*p* < 0.05, Fig. 2E). In addition, the expression level of proliferation index PCNA was significantly increased in PLC/PRF/5-STEAP3 but decreased in Hep3B-ShSTEAP3 and SNU449-ShSTEAP3 (Fig. 2F). Furthermore, to validate the in vivo effect of STEAP3 on tumor growth, a xenograft mouse model was carried out (Fig. 2G). As expected, the group of mice injected with Hep3B-ShSTEAP3 developed remarkably smaller and lighter tumor masses than that of the control group (*p* < 0.01, Fig. 2G). Nevertheless, aberrant expression of STEAP3 didn’t significantly affect cell motility and invasion as examined by our transwell assays (*p* > 0.05, Fig. 2H, Supplementary Fig. 1).

### STEAP3 modulates ERK and JNK signaling to promote stemness and cell cycle progression in HCC

**Fig. 3:**
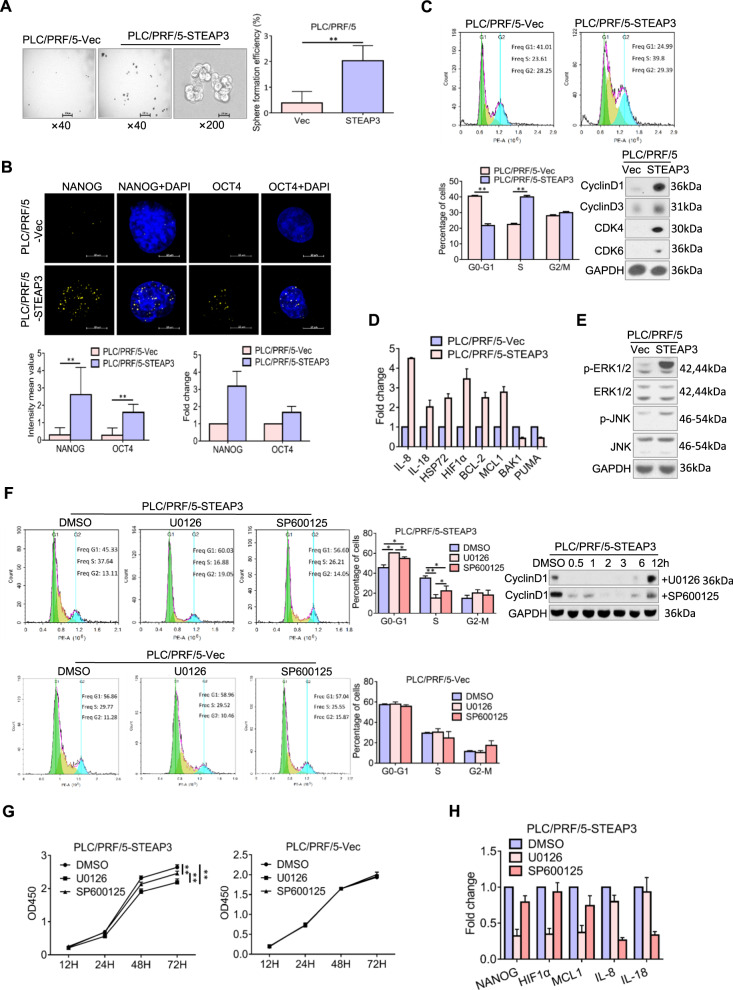
STEAP3 modulates ERK and JNK signaling to promote stemness and cell cycle progression in HCC. **A** Representative images of morphology of spheroids in PLC/PRF/5-STEAP3 and control cells were showed. Sphere formation efficiency (SFE) was calculated and presented as means ± SD of three independent experiments. Scale bar, 200px. **p < 0.01. **B** Expression levels of NANOG and OCT4 (yellow) were examined by IF in PLC/PRF/5-STEAP3 and control cells. Nuclei were visualized by DAPI (blue). Fluorescence intensity mean values were measured in 30 cells that showed strong fluorescence in each group and presented as means ± SD (left, lower panel). Scale bar, 10 μm. **p < 0.01. mRNA levels of NANOG and OCT4 were examined by real-time RT-PCR and presented as means ± SD of three independent experiments (right, lower panel). **C** Cell cycle was analyzed by flow cytometry after cells were synchronized by starvation and supplemented with serum for 8 h in PLC/PRF/5-STEAP3 and control cells (upper panel). Data are presented as means ± SD of three independent experiments. **p < 0.01 (left, lower panel). Expression levels of CyclinD1, CyclinD3, CDK4, and CDK6 were examined by western blot (right, lower panel). **D** mRNA levels of inflammatory factors (IL-8, IL-18, and HSP72), HIF1α, and BCL-2 family members (BCL-2, MCL1, BAK1, and PUMA) were examined by real-time RT-PCR and presented as means ± SD of three independent experiments. **E** Phosphorylation levels of ERK1/2 and JNK were examined by western blot in PLC/PRF/5-STEAP3 and control cells. **F** PLC/PRF/5-STEAP3 and PLC/PRF/5-Vec cells were synchronized by starvation, administrated with U0126 (10 μM, 2 h), and SP600125 (50 μM, 45 min), respectively, and supplemented with serum for 8 h. Cell cycle was analyzed by flow cytometry. Cells administrated with DMSO were used as a control. Data are presented as means ± SD of three independent experiments. *p < 0.05, **p < 0.01. Expression levels of CyclinD1 were examined by western blot in PLC/PRF/5-STEAP3. **G** Cell viability was examined by CCK8 assays in PLC/PRF/5-STEAP3, and PLC/PRF/5-Vec treated with U0126 and SP600125, respectively. Cells administrated with DMSO were used as a control. Data are presented as means ± SD of three independent experiments. **p < 0.01. **H** mRNA levels of NANOG, HIF1α, MCL1, IL-8, and IL-18, after administration with U0126 and SP600125 in PLC/PRF/5-STEAP3, were examined by real-time RT-PCR and presented as means ± SD of three independent experiments.

To reveal how aberrant nuclear expression of STEAP3 promotes HCC cells proliferation, we first examined its modulation on stemness phenotype. As compared with control cells, PLC/PRF/5-STEAP3 exhibited much higher sphere-propagating capacity (*p* < 0.01, Fig. 3A). Consistently, both protein and mRNA levels of pluripotency transcription factors, NANOG and OCT4, were elevated in PLC/PRF/5-STEAP3, reflecting that nuclear STEAP3 participated in promoting stemness phenotype (Fig. 3B). Subsequently, by monitoring cell cycle progression, we found that PLC/PRF/5-STEAP3 passed through G1 checkpoint much faster than control cells, and correspondingly, G1 Cyclins (Cyclin D1 and Cyclin D3) and CDKs (CDK4 and CDK6) levels were significantly upregulated compared with that in control cells (*p* < 0.01, Fig. 3C). In addition, we found that inflammatory factors (IL-8, IL-18, and HSP72), HIF1α, and anti-apoptotic proteins (BCL-2 and MCL1) were substantially transcriptionally upregulated by STEAP3, while pro-apoptotic proteins (BAK1 and PUMA) were downregulated (Fig. 3D).

To further clarify the intracellular signaling that mediated the pro-proliferation effect of STEAP3, we focused on MAPK pathways, which play essential roles in regulating cell survival and proliferation. We found that ERK and JNK activities were substantially upregulated, as evidenced by increased phosphorylation of ERK1/2 and JNK (Fig. 3E). Then, by adopting specific ERK and JNK inhibitors, U0126 and SP600125, we explored their individual role in cell proliferation. Inhibition on ERK and JNK, especially on ERK, significantly repressed G1/S transition (*p* < 0.05, Fig. 3F, upper panel) and cell viability (*p* < 0.01, Fig. 3G, left), while, didn’t affect cell cycle progression (*p* > 0.05, Fig. 3F, lower panel) and cell viability in PLC/PRF/5-Vec (*p* > 0.05, Fig. 3G, right). Furthermore, ERK inhibition abrogated STEAP3-induced upregulation of NANOG, HIF1α, and MCL1 transcription, while JNK inhibition only decreased IL-8 and IL-18 transcription (Fig. 3H). These results suggested that ERK signaling played a more vital part in STEAP3-enhanced cell proliferation, even though both ERK and JNK signaling contributed.

### STEAP3 regulates RAC1 to activate ERK-STAT3/JNK-STAT6 signaling axes to promote cell proliferation in HCC

**Fig. 4:**
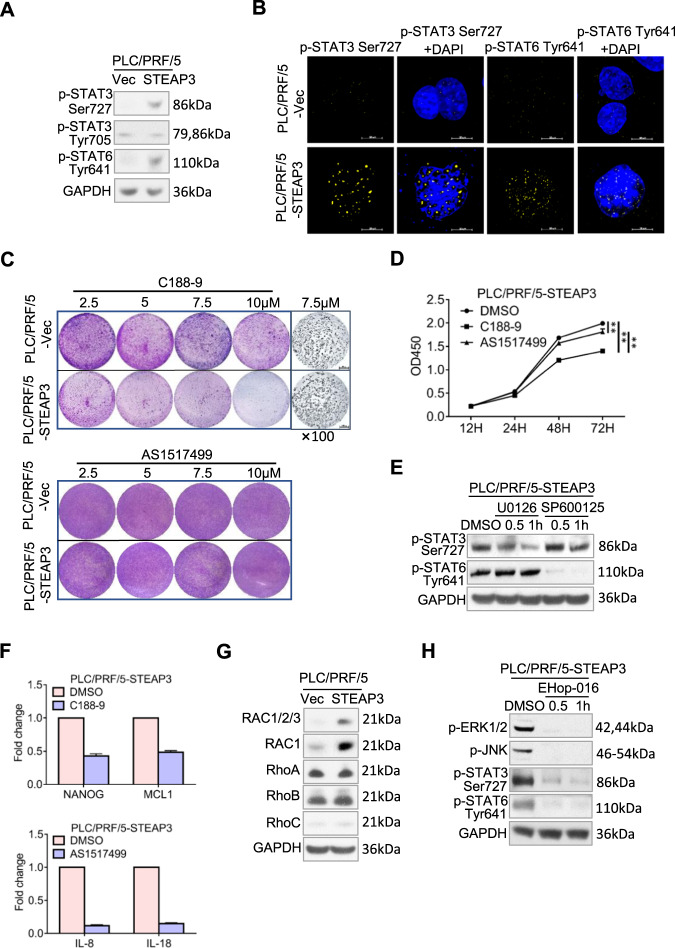
STEAP3 regulates RAC1 to activate ERK-STAT3/JNK-STAT6 signaling axes to promote cell proliferation in HCC. **A** Phosphorylation levels of STAT3 and STAT6 were examined by western blot. **B** Nuclear translocation of p-STAT3 Ser727 and p-STAT6 Tyr641 was verified by IF in PLC/PRF/5-STEAP3 and control cells. Nuclei were visualized by DAPI (blue). Scale bar, 10 μm. **C** PLC/PRF/5-STEAP3 and control cells were starved for 24 h, then supplemented with serum and administrated with different doses (2.5, 5, 7.5, 10 μM) of STAT3 and STAT6 inhibitor, C188-9 and AS1517499, respectively, for 2 h. Scale bar, 200 μm. Cells were fixed, and visualized by crystal violet. **D** Cell viability was examined by CCK8 assays in PLC/PRF/5-STEAP3 treated with C188-9 (0.5 μM) and AS1517499 (1 μM), respectively. Cells administrated with DMSO were used as a control. Data are presented as means ± SD of three independent experiments. **p < 0.01. **E** Phosphorylation levels of STAT3 Ser727 and STAT6 Tyr641 were examined by western blot after U0126 and SP600125 administration for 0.5 and 1 h, respectively, in PLC/PRF/5-STEAP3. **F** mRNA levels of NANOG, MCL1, IL-8 and IL-18, were examined by real-time RT-PCR in PLC/PRF/5-STEAP3 after administration with C188-9 and AS1517499, respectively, and presented as means ± S.D. of three independent experiments. **G** Expression levels of Rho GTPase family members (RAC1/2/3, RAC1, RhoA, RhoB, and RhoC) were examined by western blot in PLC/PRF/5-STEAP3 and control cells. **H** Phosphorylation levels of ERK1/2, JNK, STAT3 Ser727, and STAT6 Tyr641 were examined by western blot in PLC/PRF/5-STEAP3 after RAC1 inhibition by EHop-016 (5 μM) for 0.5 and 1 h.

STAT (Signal transducers and activators of transcription) is one of the most important downstream transcription factors utilized by various pro-proliferation signals. Therefore, to further reveal how STEAP3-promoted cell proliferation, we examined its regulation on STAT family members. Our results showed that STEAP3 significantly upregulated the expression levels and nuclear translocation of p-STAT3 Ser727 and p-STAT6 Tyr641 (Fig. 4A, B). To verify to what extent did they individually participate in STEAP3-induced signaling, we adopted specific STAT3 and STAT6 inhibitors, C188-9, and AS1517499, respectively, to block their activation. As compared to control cells, STAT3 inhibition led to much more massive cell death under starvation-induced stress in PLC/PRF/5-STEAP3 (Fig. 4C, upper panel), while STAT6 inhibition barely affected cell survival (Fig. 4C, lower panel). Furthermore, STAT3 inhibition decreased cell viability to a greater extent than STAT6 inhibition (*p* < 0.01, Fig. 4D). These results strongly indicated that STAT3 played a more important part in modulating cell proliferation induced by STEAP3.

Subsequently, by utilizing U0126 and SP600125, we found that ERK inhibition significantly decreased the expression of p-STAT3 Ser727, while JNK inhibition blocked STAT6 activation, suggesting that ERK-STAT3, and JNK-STAT6 signaling axes participated in mediating STEAP3-promoted cell proliferation (Fig. 4E). Consistently, STAT3 inhibition decreased NANOG and MCL1 transcription, while STAT6 inhibition reduced the transcription of IL-8 and IL-18 (Fig. 4F).

The Rho family of small GTPases acts as molecular switches to regulate the activation of signaling pathways, including MAPK pathways. Hence, we examined the expression levels of the members of this family and found that RAC1 was considerably upregulated by STEAP3 (Fig. 4G). Through inhibiting RAC1 by a specific inhibitor, EHop-016, we further established that RAC1 was the upstream activator of ERK-STAT3/JNK-STAT6 signaling, in light of that these signaling was notably abrogated by EHop-016 administration (Fig. 4H).

### STEAP3 facilitates nuclear trafficking of EGFR to enhance STAT3 transactivity

**Fig. 5:**
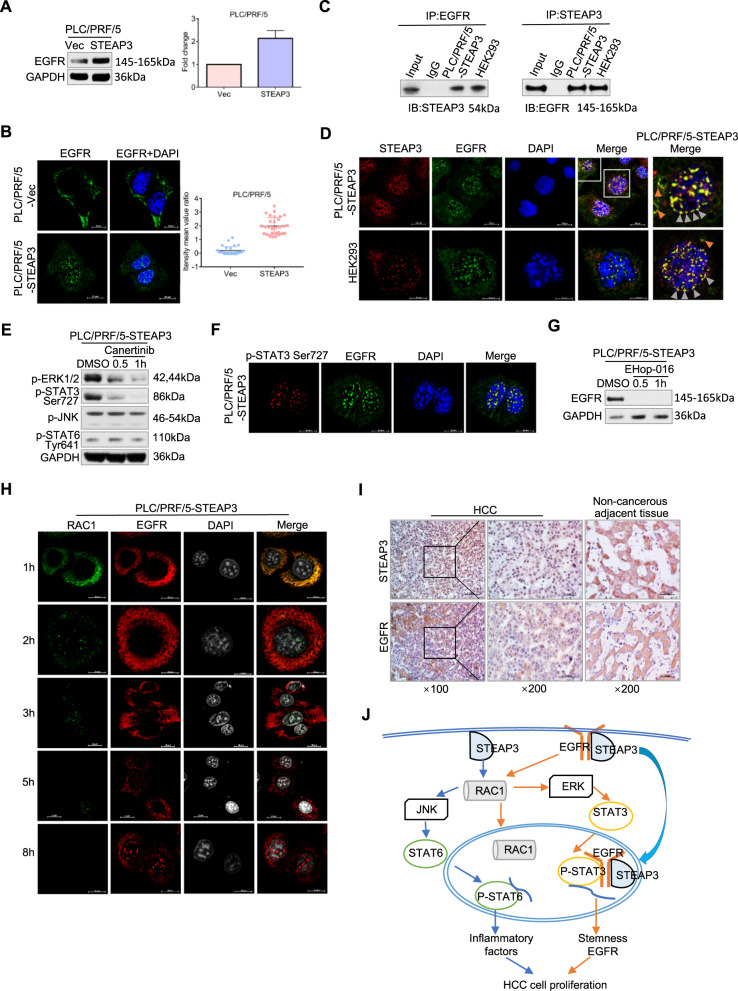
STEAP3 facilitates nuclear trafficking of EGFR to enhance STAT3 transactivity. **A** Protein and mRNA levels of EGFR were examined by western blot (left) and real-time RT-PCR (right), respectively, in PLC/PRF/5-STEAP3 and control cells. Data are presented as means ± SD of three independent experiments. **B** PLC/PRF/5-STEAP3 and control cells were synchronized by starvation and supplemented with serum for 8 h. Subcellular distribution of EGFR was examined by IF, and the fluorescence intensity mean values of that were measured in randomly chose 30 cells (left). Scale bar, 10 μm and 20 μm. Intensity means value ratios were determined by comparing the intensity mean values in the nuclei with that in the cytoplasm (right). *p* < 0.01. **C** Interaction between STEAP3 and EGFR was analyzed in PLC/PRF/5-STEAP3 and HEK293 by Co-IP assay. **D** Co-localization of STEAP3 (red) with EGFR (green) was verified by confocal microscope in PLC/PRF/5-STEAP3 (left, upper panel) and HEK293 (left, lower panel). Magnified images with merged STEAP3 and EGFR fluorescence signals in PLC/PRF/5-STEAP3 were shown (right). Nuclei were visualized by DAPI (blue). Scale bar, 10 μm and 20 μm. **E** Phosphorylation levels of ERK1/2, JNK, STAT3 Ser727, and STAT6 Tyr641 were examined by western blot in PLC/PRF/5-STEAP3 after EGFR inhibition using Canertinib (5 μM) for 0.5 and 1 h. Cells administrated with DMSO were used as a control. **F** Co-localization of p-STAT3 Ser727 (red) with EGFR (green) was examined by confocal microscope in PLC/PRF/5-STEAP3. Nuclei were visualized by DAPI (blue). Scale bar, 10 μm. **G** Expression level of EGFR was examined by western blot in PLC/PRF/5-STEAP3 after EHop-016 (5 μM) administration for 0.5 and 1 h, respectively. Cells administrated with DMSO were used as a control. **H** PLC/PRF/5-STEAP3 cells were synchronized by starvation and supplemented with serum for indicated periods of time (1, 2, 3, 5, and 8 h), respectively. Subcellular distributions of RAC1 (green) and EGFR (red) were examined by confocal microscope. Nuclei were visualized by DAPI (dark gray). Scale bar, 10 μm and 20 μm. **I** Expression levels of STEAP3 and EGFR were examined by IHC on HCC and paired non-cancerous adjacent tissues. Representative images showed that STEAP3 and EGFR were overexpressed in the nuclei in HCC cells compared with that in non-cancerous adjacent tissues. Scale bar, 100 μm. **J** Graphic abstract depicting a proposed model for a major mechanism of STEAP3 in promoting HCC cell proliferation.

Growth factor receptors, which have long been recognized as oncogenic proteins, are the most common activators of MAPK signaling and are involved in cancer growth/progression. We found that EGFR (epidermal growth factor receptor, ErbB-1/HER-1) was significantly upregulated by STEAP3 on both protein and mRNA levels (Fig. 5A). Similar to STEAP3, we observed enhanced nuclear translocation of EGFR in PLC/PRF/5-STEAP3, compared with control cells (*p* < 0.01, Fig. 5B). Then, a clear interaction between STEAP3 and EGFR was validated in both PLC/PRF/5-STEAP3 and HEK293 (Fig. 5C, D). In addition, STEAP3 complexed with EGFR in the nuclear membrane (Fig. 5D, right, gray triangle) and nucleoplasm, besides in the cytoplasm (Fig. 5D, right, orange triangle), in PLC/PRF/5-STEAP3, implicating that STEAP3 participated in facilitating nuclear trafficking of EGFR. Furthermore, STEAP3-induced activation of ERK and STAT3, not that of JNK, was substantially abrogated by EGFR inhibition (Fig. 5E). In agreement with reports that nuclear EGFR requires co-factors, such as STAT3 [20], to transactivate target genes, the interaction between EGFR and p-STAT3 Ser727 was found in PLC/PRF/5-STEAP3 (Fig. 5F). These results clearly indicated that STEAP3 not only facilitated the nuclear trafficking of EGFR but also complexed with it in the nucleus, to enhance the transactivity of STAT3.

Since RAC1 was increasingly translocated to the nucleus in cancer cells [21], we subsequently examined the spatio-temporal coordination of EGFR with RAC1. As our results showed, RAC1 inhibition significantly decreased the expression level of EGFR in PLC/PRF/5-STEAP3 (Fig. 5G). The great magnitude of G1/S transition was completed in the first 8 h of a cell cycle in PLC/PRF/5-STEAP3 (Fig. 3C). During this period of time, RAC1 and EGFR first co-localized in the cytoplasm, then nuclear translocation of RAC1 preceded and peaked before nuclear trafficking of EGFR, suggesting that RAC1 might exert other functions besides molecular switch in PLC/PRF/5-STEAP3 (Fig. 5H). Then, a clear co-localization between RAC1 and Lamin A/C, which is a nuclear structural component mainly found in the nuclear lamina, was observed (Supplementary Fig. 2). These results indicated that RAC1 might participate in regulating nuclear structure to facilitate EGFR nuclear trafficking.

Furthermore, we evaluated the potential correlations between the nuclear expression of STEAP3 and EGFR by IHC in our cohort of 200 HCC tissues (Fig. 5I). Our results showed that the nuclear expression of STEAP3 was positively correlated with that of EGFR (*p* < 0.001, Chi-square test, Table 3).

**Table 3:** Correlation between nuclear expression of STEAP3 and EGFR in hepatocellular carcinomas.

	Nuclear EGFR expression			
	Low	High	Total	*P*-value^*^
**Nuclear STEAP3 expression**				
Low	100 (88.5%)	13 (11.5%)	113 (56.5%)	0.000
High	38 (43.7%)	49 (56.3%)	87 (43.5%)	
Total	138 (69.0%)	62 (31.0%)	200	

^*^Chi-square test.

Together, these results suggested that STEAP3 initiated a positive feedback loop that acted through EGFR (EGFR, RAC1, ERK) to modulate the transactivity of STAT3.

## Discussion

Overexpression of STEAP3 has been proved to be involved in tumor progression and predicts poor prognoses in several human cancers [13–15]. However, the molecular mechanisms underlying its oncogenic role are largely unknown. In the present study, unprecedently, we observed that STEAP3 was aberrantly overexpressed in the nuclei of HCC cells, which positively associated with tumor differentiation and poor prognosis. Using a series of in vitro and in vivo assays, we provided evidence that nuclear STEAP3 significantly enhances HCC cells proliferation by promoting stemness phenotype and cell cycle progression via upregulating the expression and nuclear trafficking of EGFR and modulating EGFR-RAC1-ERK-STAT3 and RAC1-JNK-STAT6 signaling axes. These results strongly support the oncogenic role of STEAP3 in the progression of HCC, and it could be an independent prognostic marker to predict patients’ outcomes.

Previous studies showed that STEAP3 localizes in the plasma membrane, near the nucleus, and in vesicular tubular structures [10]. It is upregulated upon p53 activation and involved in promoting apoptosis and cell cycle delay in the G2/M phase by interacting with Nix and Myt1 [6, 11, 12]. In addition, enhanced STEAP3 expression facilitates and promotes the vesicular secretion of TCTP, a tubulin-binding protein that participates in regulating cytoskeleton assemble and elongation machinery and inhibiting apoptosis [22, 23]. In the liver, the expression level of STEAP3 was reported to be substantially diminished in HCC nodules compared with cirrhotic peritumoral tissues and is dependent on tumor differentiation stage, with lower levels of STEAP3 associated with moderately or poorly differentiated tumors [18, 19]. Nevertheless, in our present study, for the first time, an aberrant nuclear expression pattern of STEAP3 was observed, which correlated with tumor differentiation and predicted poor prognosis, supporting its oncogenic role in the progression of HCC. Furthermore, enhanced nuclear expression of STEAP3 promotes cancer cell proliferation by enhancing stemness phenotype and accelerating G1/S transition through regulating intracellular signaling and facilitating nuclear trafficking of EGFR.

Nuclear translocation of EGFR was first found in hepatocytes that underwent regeneration [24]. Nuclear EGFR correlates with poor outcomes in multiple human malignancies, including breast cancer [25], ovarian cancer [26], non-small cell lung cancer (NSCLC) [27], and oropharyngeal squamous carcinoma [28]. In addition, the nuclear localization of EGFR is responsible for resistance to EGFR-targeted therapies and cisplatin [29–31]. Due to lack of a putative DNA binding domain, activated nuclear EGFR act as a transcriptional co-factor to modulate certain target genes, such as iNOS, Aurora-A, c-Myc, and B-Myb, through association with transcriptional factors that harbor DNA binding capability, including STAT3 [20, 32–34]. However, the subcellular trafficking mechanism of EGFR hasn’t been fully explored yet. Previous study showed that importin-β and CRM1 form a complex with EGFR to facilitate its nuclear translocation [35]. In this study, we reveal that STEAP3 facilitated translocation as a new mechanism of EGFR nuclear trafficking in HCC cells. In the nucleus, STEAP3 and EGFR together enhance the transactivity of STAT3. In addition to HCC cells, similar interaction between STEAP3 and EGFR was observed in HEK293, suggesting it might be a common functional role of STEAP3 in human cancers.

In the present study, we found that STEAP3 modulates EGFR-ERK-STAT3 and JNK-STAT6 signaling to promote HCC cells proliferation. Constitutive activation of STAT family members, which possess properties of oncogenes, has been demonstrated in several human malignancies [36]. Increased activity of STAT3 induced by EGFR has been found in breast, prostate, lung, head, and neck, pancreatic and colon cancer [36–40]. STAT6 is also constitutively activated and involved in tumor initiation and progression [41–43]. Persistent activation of STAT6 is responsible for promoting the local pro-inflammatory response to favor the development of colitis-associated colon cancer [42]. Ser707 phosphorylation of STAT6 by JNK was reported to play a role in crosstalk between the intracellular signals of IL-4 and IL-1β by negatively regulating IL-4-induced transcriptional activation of STAT6 [44]. However, STAT6 possesses five potential phosphorylation sites that match the consensus substrate sequences of JNK, implying that JNK-STAT6 signaling might exert different functions in a context-dependent way. In this study, STEAP3 activated JNK to upregulate Tyr641 phosphorylation of STAT6 to promote transcription of IL-8 and IL-18, which have pro-tumoral functions through regulating angiogenesis, survival signaling, and immunosuppression [45, 46].

RAC1, a member of the Rho family of small GTPases that acts as molecular switch through GDP-bound inactive and GTP-bound active states and regulates a wide range of cellular processes, including cell proliferation, differentiation, survival, motility, nuclear and cytoskeleton assembly, was recently reported that its nuclear translocation associates with carcinogenesis and enhanced aggressiveness of cancer. As a signaling hub integrating receptor-associated intracellular signaling into dynamic cellular response, RAC1 has been shown to be a critical component of EGFR signaling in various tumors and implicated in regulating ERK/JNK signaling [47, 48]. EGFR stimulated ERK activation phosphorylates RAC1 on Thr108 and targets RAC1 for nuclear translocation [49]. Nuclear RAC1 could modulate nuclear shape and position in an actin-dependent fashion and alter nuclear membrane fluidity and order [50, 51]. Therefore, as we observed in our study, nuclear importing of RAC1 prior to that of EGFR after their interaction in the cytoplasm and its interaction with Lamin A/C might change the nucleus to be more accessible to EGFR trafficking.

In summary, our study revealed, for the first time, the nuclear expression pattern of STEAP3 in human HCC tissues. Our results support the concept that up-regulated nuclear expression of STEAP3 plays an important role in the acquisition of a poor prognostic phenotype in HCC. In addition, functional and mechanistic studies suggest that STEAP3 participates in promoting HCC cell proliferation through modulating nuclear trafficking of EGFR and the activities of EGFR-RAC1-ERK-STAT3 and RAC1-JNK-STAT6 axes, which might be contributed, at least in part, to the progression of human HCC.

## Supplementary information

Supplementary

Supplementary Fig 1

Supplementary Fig 2

## Data Availability

The data that support the findings of this study are available from the corresponding author upon reasonable request.
