# Supplementary material for: STEAP3 promotes cancer cell proliferation by facilitating nuclear trafficking of EGFR to enhance RAC1-ERK-STAT3 signaling in hepatocellular carcinoma
Source: Cell Death Dis. 2021 Nov 5;12(11):1052. doi: 10.1038/s41419-021-04329-9 (PMC8571373; doi:10.1038/s41419-021-04329-9)
Supplement: Supplementary file 1 — Supplementary [file 41419_2021_4329_MOESM1_ESM.docx]

**STEAP3 promotes cancer cell proliferation by facilitating nuclear trafficking of EGFR to enhance RAC1-ERK-STAT3 signaling in hepatocellular carcinoma**

Li-Li Wang, Jie Luo, Zhang-Hai He, Ye-Qing Liu, Hai-Gang Li, Dan Xie and

Mu-Yan Cai

Table of contents

Supplementary Tables………………………………………………………………..2

Supplementary Material and Methods……………………………………………….6

Supplementary Figure legends……………………………………………………….7

**Supplementary Table 1. Primary antibodies used in this study**

| **Name** | **Supplier** | **Cat no.** | **Application** | **Dilution** |
| --- | --- | --- | --- | --- |
| STEAP3 | Abcam | ab151566 | IF  Western blot | 1:300  1:5000 |
| STEAP3 | Proteintech | 60199-1-Ig | IP  IHC | 1:50  1:500 |
| p-ERK1/2 | Cell Signaling Technology | 4370 | Western blot | 1:100  1:5000 |
| p-JNK | Cell Signaling Technology | 4668 | Western blot | 1:10000 |
| p-STAT3 (Ser727) | Cell Signaling Technology | 9134 | Western blot | 1:1000 |
| p-STAT3 (Ser727) | Cell Signaling Technology | 34911 | IF | 1:800 |
| p-STAT3 (Tyr705) | Cell Signaling Technology | 9145 | Western blot | 1:1000 |
| p-STAT6  (Tyr641) | Cell Signaling Technology | 56554 | IF  Western blot | 1:400  1:1000 |
| RAC1/2/3 | Cell Signaling Technology | 2465 | Western blot | 1:1000 |
| RAC1 | Abcam | ab155938 | Western blot | 1:1000 |
| Cdc42 | Cell Signaling Technology | 2466 | Western blot | 1:1000 |
| RhoA | Cell Signaling Technology | 2177 | Western blot | 1:1000 |
| RhoB | Cell Signaling Technology | 2098 | Western blot | 1:1000 |
| RhoC | Cell Signaling Technology | 3430 | Western blot | 1:1000 |
| NANOG | Cell Signaling Technology | 4903 | IF | 1:300 |
| OCT4 | Cell Signaling Technology | 2750 | IF | 1:200 |
| EGFR | Proteintech | 66455-1-Ig | IF | 1:500 |
| EGFR | Proteintech | 18986-1-AP | Western blot  IP  IHC | 1:500  1:50  1:500 |
| PCNA | Cell Signaling Technology | 13110 | Western blot | 1:10000 |
| GAPDH | Proteintech | 60004-1-Ig | Western blot | 1:1000 |
| CDK6 | Cell Signaling Technology | 13331 | Western blot | 1:1000 |
| CDK4 | Cell Signaling Technology | 12790 | Western blot | 1:1000 |
| Cyclin D1 | Cell Signaling Technology | 2978 | Western blot | 1:5000 |
| Cyclin D3 | Cell Signaling Technology | 2936 | Western blot | 1:5000 |
| Lamin A/C | Cell Signaling Technology | 4777 | IF | 1:200 |

**Supplementary Table 2. Chemicals used in this study**

| **Name** | **Supplier** | **Cat no.** | **Concentration** |
| --- | --- | --- | --- |
| U0126 | Cell Signaling Technology | 9903 | 10 μM |
| SP600125 | Cell Signaling Technology | 8177 | 50 μM |
| C188-9 | Selleck | S8605 | 0.5-10 μM |
| AS1517499 | Selleck | S8685 | 1-10 μM |
| EHop-016 | Selleck | S7319 | 5 μM |
| Canertinib | Selleck | S1019 | 5 μM |

**Supplementary Table 3. Primers used in this study**

| Gene | Forward Primer | Reverse Primer |
| --- | --- | --- |
| NANOG | CAGCCCCGATTCTTCCACCAGTCCC | CGGAAGATTCCCAGTCGGGTTCACC |
| OCT4 | GACAGGGGGAGGGGAGGAGCTAGG | CTTCCCTCCAACCAGTTGCCCCAAAC |
| IL-8 | TTTTGCCAAGGAGTGCTAAAGA | AACCCTCTGCACCCAGTTTTC |
| IL-18 | TCTTCATTGACCAAGGAAATCGG | TCCGGGGTGCATTATCTCTAC |
| HSP72 | GCGAGGCGGACAAGAAGAA | GATGGGGTTACACACCTGCT |
| HIF1α | GAACGTCGAAAAGAAAAGTCTCG | CCTTATCAAGATGCGAACTCACA |
| BCL-2 | GGTGGGGTCATGTGTGTGG | CGGTTCAGGTACTCAGTCATCC |
| MCL1 | TGCTTCGGAAACTGGACATCA | TAGCCACAAAGGCACCAAAAG |
| BAK1 | CATCAACCGACGCTATGACTC | GTCAGGCCATGCTGGTAGAC |
| PUMA | GACCTCAACGCACAGTACGAG | AGGAGTCCCATGATGAGATTGT |
| EGFR | CCCACTCATGCTCTACAACCC | TCGCACTTCTTACACTTGCGG |

**Supplementary Material and Methods**

**Transwell invasion assay**

Transwell chambers with 8-μm pore polycarbonate membranes (Corning, NY, USA) were coated with 100 μl matrix gel (Corning, NY, USA) diluted according to the manufacturer’s instructions and incubated overnight. Cells were starved with serum-free medium for 24h and seeded at a density of 10^5^ in the upper chamber. Medium containing 20% FBS was added to the lower chambers. After incubating for 36h, cells were stained with crystal violet. Cells crossed through the pores to the lower surface of the filter were counted in 20 high-power fields (200×) under a microscope.

Supplementary Figure Legends

Supplementary Figure 1:

Cell invasion was determined by Transwell invasion assays. Data are showed as means±S.D. of three independent experiments. Scale bar, 200μm. *p* > 0.05.

Supplementary Figure 2:

PLC/PRF/5-STEAP3 cells were synchronized by starvation and supplemented with serum for 2h. Co-localization of RAC1 (green) and Lamin A/C (red) was examined by confocal microscope. Nuclear was visualized by DAPI (dark grey). Scale bar, 10μm.
