# Supplementary figures and images for: STEAP3 promotes cancer cell proliferation by facilitating nuclear trafficking of EGFR to enhance RAC1-ERK-STAT3 signaling in hepatocellular carcinoma
Source: Cell Death Dis. 2021 Nov 5;12(11):1052. doi: 10.1038/s41419-021-04329-9 (PMC8571373; doi:10.1038/s41419-021-04329-9)

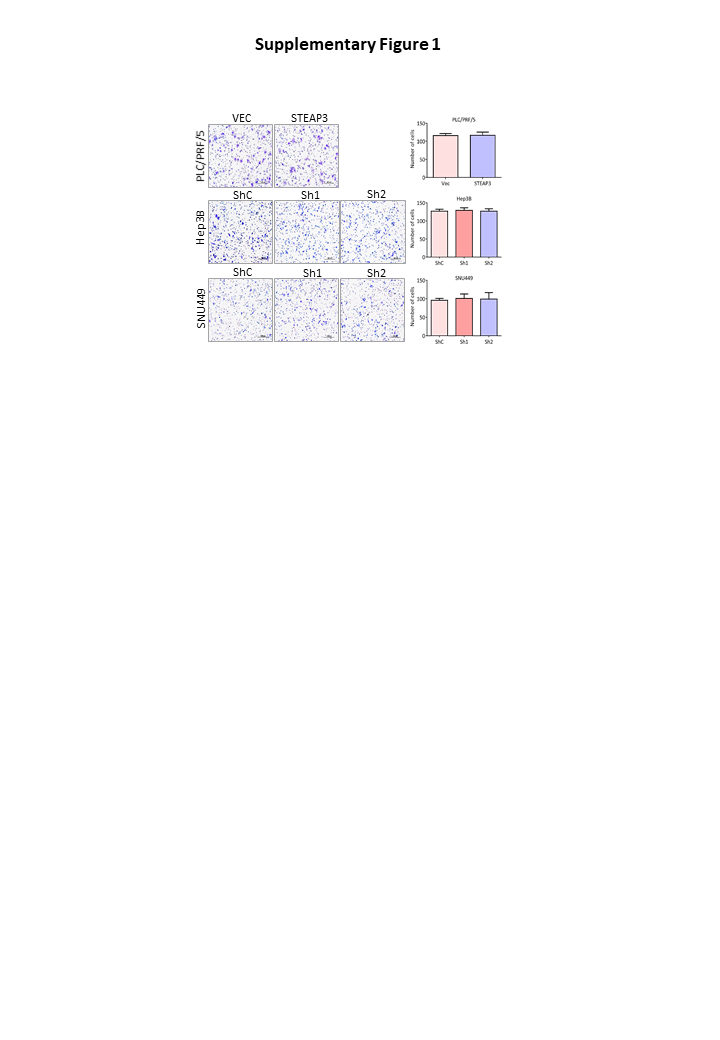

Supplement: Supplementary file 2 — Supplementary Fig 1 [file 41419_2021_4329_MOESM2_ESM.png]

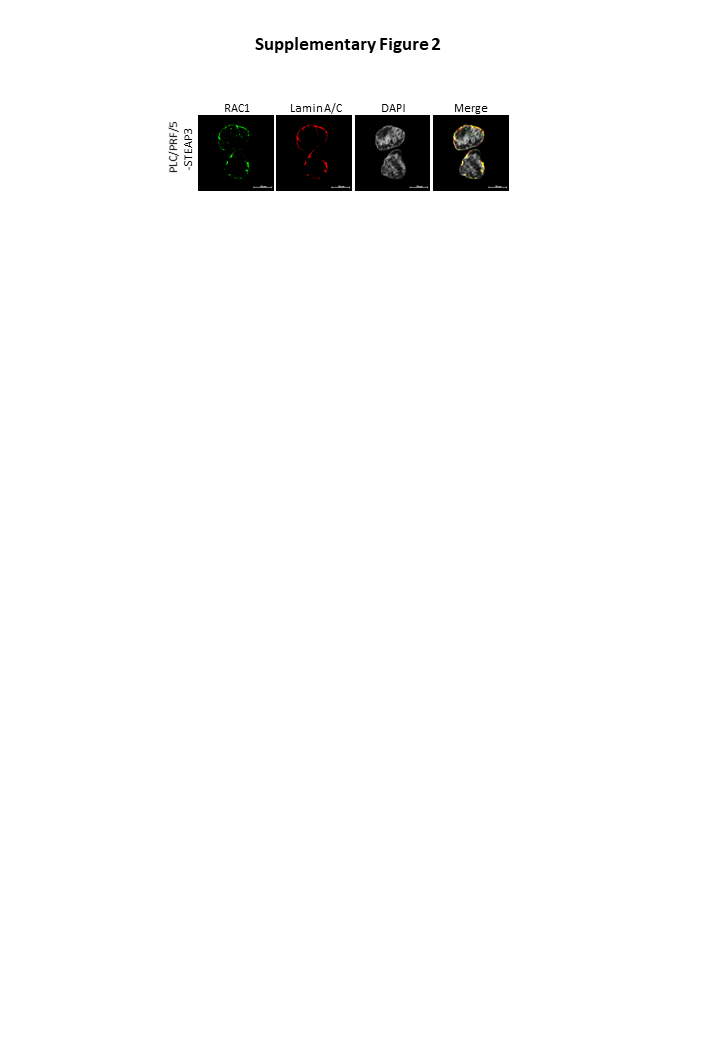

Supplement: Supplementary file 3 — Supplementary Fig 2 [file 41419_2021_4329_MOESM3_ESM.png]
